# Prognostic impact of programmed cell death ligand 1 (PD-L1) expression and its association with epithelial-mesenchymal transition in extrahepatic cholangiocarcinoma

**DOI:** 10.18632/oncotarget.25050

**Published:** 2018-04-13

**Authors:** Takashi Ueno, Takahiro Tsuchikawa, Kanako C. Hatanaka, Yutaka Hatanaka, Tomoko Mitsuhashi, Yoshitsugu Nakanishi, Takehiro Noji, Toru Nakamura, Keisuke Okamura, Yoshihiro Matsuno, Satoshi Hirano

**Affiliations:** ^1^ Department of Gastroenterological Surgery II, Division of Surgery, Faculty of Medicine, Graduate School of Medicine, Hokkaido University, Sapporo, Hokkaido, Japan; ^2^ Department of Surgical Pathology, Hokkaido University Hospital, Sapporo, Hokkaido, Japan

**Keywords:** extrahepatic cholangiocarcinoma, PD-L1, tumor infiltrating lymphocytes, epithelial-mesenchymal transition, immunohistochemical analysis

## Abstract

Extrahepatic cholangiocarcinoma (eCCA) has a poor prognosis. Although the possibility of immunotherapy has been studied, immune checkpoint molecules such as programmed death ligand 1 (PD-L1) in eCCA are not well understood. Epithelial-mesenchymal transition (EMT) has recently been shown to regulate PD-L1 expression. Our aims were to assess the clinicopathological significance of tumor-infiltrating lymphocytes (TILs) and tumor PD-L1 expression in eCCA and to compare these immune responses with EMT marker expression. In this retrospective study, we conducted immunohistochemical analyses for 117 patients with eCCA. We stained for CD4, CD8, Foxp3, and PD-L1 as markers reflecting local immune responses, and for E-cadherin, N-cadherin, vimentin, ZEB1, ZEB2, SNAIL, and TWIST as markers associated with EMT. High numbers of CD4+ and CD8+ TILs correlated with node-negative (*P* = 0.009 and *P* = 0.046, respectively) and low SNAIL expression (*P* = 0.016 and *P* = 0.022, respectively). High PD-L1 expression was associated with poor histopathological classification (*P* = 0.034), and low E-cadherin (*P* = 0.001), high N-cadherin (*P* = 0.044), high vimentin (*P* < 0.001) and high ZEB1 (*P* = 0.036) expression. Multivariate analysis showed that CD4+ TILs, PD-L1 expression and N-cadherin expression were independent prognostic factors (hazard ratio (HR) = 0.61; 95% confidence interval (CI) = 0.38–1.00; HR=4.27; 95% CI = 1.82–9.39; HR = 2.20; 95% CI = 1.18–3.92, respectively). These findings could help to identify potential biomarkers for predicting not only the prognosis, but also the therapeutic response to immunotherapy for eCCA.

## INTRODUCTION

Extrahepatic cholangiocarcinoma (eCCA) arises from the epithelium of the main bile duct [1]. The incidence of this rare disease has been reported as 1–2 cases per 100,000 per year [2]. Although surgical resection is the only curative option for eCCA, many patients develop recurrent disease. Overall, it has been reported that 5-year survival rates after resection range from 20% to 40% [2, 3]. Development of multimodal treatment strategies is therefore important [4–7].

Recent research has focused on the role which the immunological tumor microenvironment plays in the regulation of tumor growth. Tumor-infiltrating lymphocytes (TILs) such as CD4+ and CD8+ T lymphocytes play important roles in host immune response. The level of local infiltration of CD4+ and CD8+ T lymphocytes has been reported as a good prognostic factor in several cancers, including biliary tract cancer [8–13]. However, the host immune system shifts from an initial tumor elimination phase to a subsequent escape phase that enables tumor cells to escape or survive the anti-tumor immune response [14]. Additionally, regulatory T cells (Tregs) in the tumor microenvironment may have a negative impact on immune responses within the tumor microenvironment [15]. The interaction between programmed death ligand 1 (PD-L1) and programmed death 1 (PD-1) receptor has recently been identified as a mechanism for the regulation of cytotoxic immune responses by activated T cells [16]. This mechanism has been highlighted and investigated in terms of associations with the therapeutic effects of immune checkpoint inhibitors, which block interactions between PD-L1 and PD-1, in the treatment of various cancers [17]. PD-L1 expression on tumor cells has been reported as a poor prognostic factor in various cancers [18–20].

PD-L1 expression on tumor cells can result in constitutive oncogenic signaling and responsiveness to the inflammatory signals produced by active tumor-infiltrating immune cells [21, 22]. Furthermore, in addition to the above mechanism of PD-L1-PD-1 interaction, recent studies have suggested that epithelial-mesenchymal transition (EMT), a process that causes transformation of epithelial cells into mesenchymal cells [23], correlates with the cell signaling associated with PD-L1 expression and subsequent suppression of TILs [24–28]. We have previously reported that the mesenchymal phenotype, which expresses mesenchymal proteins such as N-cadherin and vimentin, [23, 29] might be related to poorer prognosis in eCCA [30].

Although there have been two reports related to the prognostic significance of PD-L1 expression in eCCA, the significance of PD-L1 expression and its association with EMT remain unclear [31, 32] due to the limitation of several biases for preoperative treatment, statistical methods of univariate analysis and small patient numbers in those studies.

The aim of this study was to clarify the prognostic impact of TILs and the expression of immune escape molecules in eCCA. Moreover, we determined the association of these immune responses with EMT-related marker expression in eCCA.

## RESULTS

### Patient clinicopathological characteristics

Surgical specimens were obtained from 117 patients with a median age of 71 years (range, 44–87 years). The median follow-up period was 27 months (range, 0–189 months) and 98 patients (84%) died during follow-up. Of the patients who died, 31 patients (26%) died of hepatic recurrence, 27 patients (23%) of peritonitis carcinomatosa, 21 patients (18%) of local recurrence and 15 patients (13%) of lymph node metastasis. No patient received chemotherapy before surgery. Table 1 shows the patients’ clinicopathological characteristics.

**Table 1:** The clinicopathological features of the 117 patients with eCCA

		n	%
Sex	Male	93	79
Age, years	≥71	59	50
Tumor size, cm	≥3	29	25
Location	Perihilar	70	60
	Distal	47	40
Histopathological classification	pap	12	10
	well	22	19
	mod	59	50
	por	24	21
Invasion to hepatic artery	Positive	5	4.3
Invasion to portal vein	Positive	24	21
Lymphatic vessel invasion	Positive	82	70
Venous invasion	Positive	75	64
Perineural invasion	Positive	102	87
pT	1	49	42
	2	14	12
	3	34	29
	4	20	17
pN	0	63	54
	1	54	46
pM	0	115	98
	1	2	1.7
pStage	I	44	38
	II	19	16
	III	33	28
	IV	21	18

### Levels of TILs in the invasive front of cancer stroma

**Figure 1:**
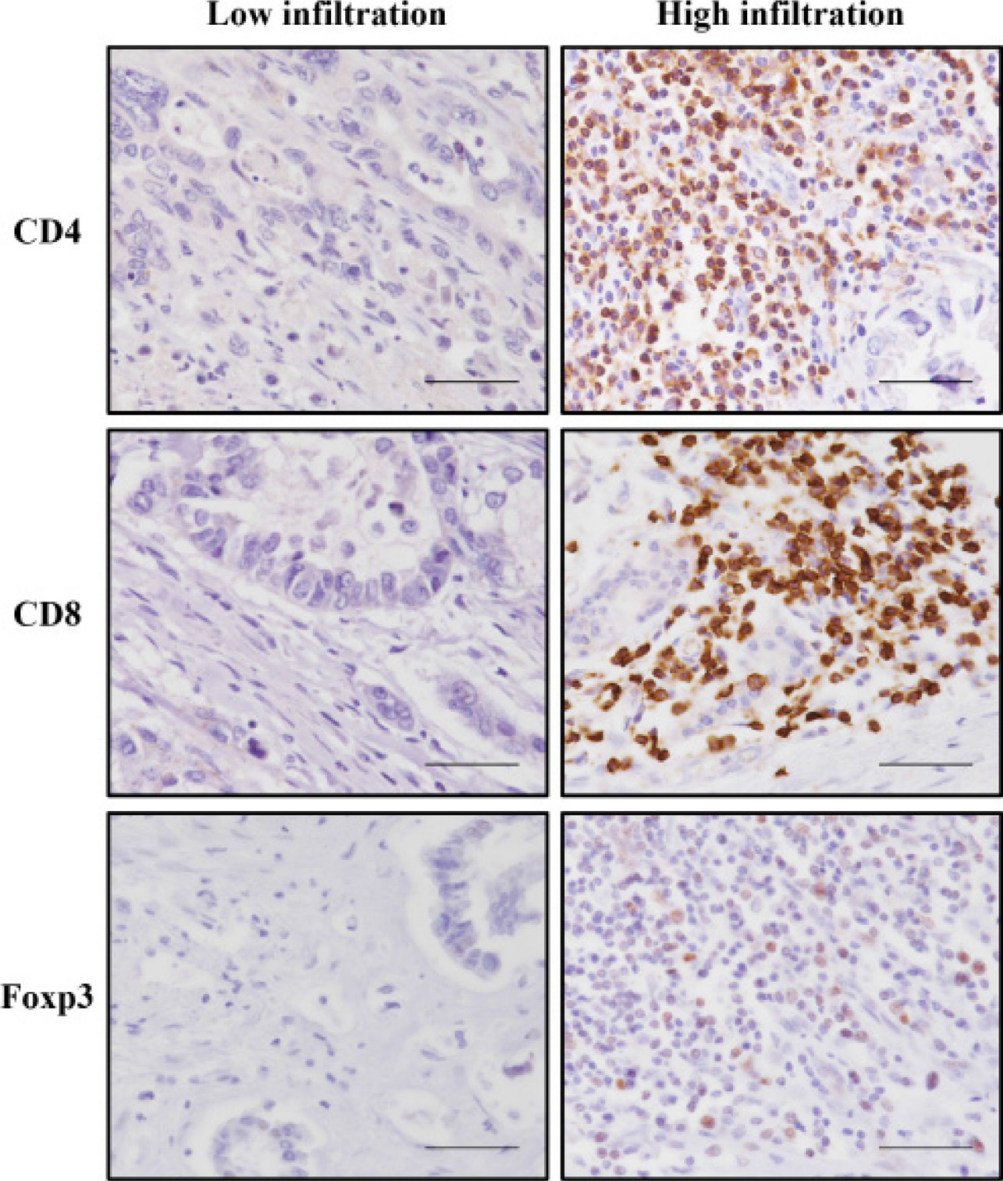
Representative immunohistochemical staining of CD4, CD8, and Foxp3 T lymphocytes that had infiltrated into the invasive front of tumor cells Each image is from a different patient. All of the figures are the same magnification (×400). Scale bar, 50 μm.

Using immunohistochemical analysis, we counted the numbers of CD4+, CD8+, and Foxp3+ T lymphocytes both in the invasive front and tumor bulk (Figure 1; Supplementary Table 1 ). No significant differences were evident between the two areas in terms of CD4+, CD8+ or Foxp3+ TILs. Furthermore, multivariate analysis showed that higher numbers of CD4+ TILs in the invasive front of cancer stroma were significantly associated with higher survival rate (Supplementary Table 2 ). These analyses showed that evaluation of TILs in the invasive front of cancer stroma reflected prognosis better than evaluation of those in the tumor bulk. Subsequent analyses regarding TILs were performed by evaluations of the invasive front of cancer stroma. Numbers of CD4+, CD8+ and Foxp3+ T lymphocytes in the invasive front of cancer stroma ranged between 2 and 384/4 high-powered fields (HPF) (median, 77/4 HPF; interquartile range, 32–136/4 HPF), between 1 and 212/4 HPF (median, 52/4 HPF; interquartile range, 26–97/4 HPF) and between 0 and 465/4 HPF (median, 9/4 HPF; interquartile range, 2–21/4 HPF), respectively. The correlation between the level of TILs and clinicopathological factors is shown in Table 2. High infiltration of CD4+ T lymphocytes correlated with well-differentiated classification (*P* = 0.028) and negative for lymph node metastasis (*P* = 0.009). High infiltration of CD8+ T lymphocytes also correlated with negative results for lymph node metastasis (*P* = 0.046).

**Table 2:** The association between TILs such as CD4+, CD8+ and Foxp3+ T lymphocytes and clinicopathological features in eCCA

		Infiltration of CD4+ T lymphocytes			Infiltration of CD8+ T lymphocytes			Infiltration of Foxp3+ T lymphocytes		
		High (*n* = 87)	Low (*n* = 30)	*P*	High (*n* = 45)	Low (*n* = 72)	*P*	High (*n* = 5)	Low (*n* = 112)	*P*
Sex	Male	70 (80%)	23 (77%)	0.66	58 (81%)	35 (78%)	0.72	4 (80%)	89 (79%)	0.98
Age, years	≥71	46 (53%)	13 (43%)	0.37	39 (54%)	20 (44%)	0.31	4 (80%)	55 (49%)	0.18
Tumor size, cm	≥3	20 (23%)	9 (30%)	0.44	17 (24%)	12 (27%)	0.71	1 (20%)	28 (25%)	0.80
Location	Perihilar	53 (61%)	17 (57%)	0.68	43 (60%)	27 (60%)	0.98	3 (60%)	67 (60%)	0.99
	Distal	34 (39%)	13 (43%)		29 (40%)	18 (40%)		2 (40%)	45 (40%)	
Histopathological classification	pap + well	30 (34%)	4 (13%)	**0.028**	22 (31%)	12 (27%)	0.65	0 (0.0%)	34 (30%)	0.14
	mod + por	57 (66%)	26 (87%)		50 (69%)	33 (73%)		5 (100%)	78 (70%)	
Invasion to hepatic artery	Positive	5 (5.8%)	0 (0.0%)	0.18	3 (4.2%)	2 (4.4%)	0.94	0 (0.0%)	5 (4.5%)	0.63
Invasion to portal vein	Positive	17 (20%)	7 (23%)	0.66	12 (17%)	12 (27%)	0.19	2 (40%)	22 (20%)	0.27
Lymphatic vessel invasion	Positive	59 (68%)	23 (77%)	0.36	48 (67%)	34 (76%)	0.31	3 (60%)	79 (71%)	0.61
Venous invasion	Positive	53 (61%)	22 (73%)	0.22	43 (60%)	32 (71%)	0.21	3 (60%)	72 (64%)	0.85
Perineural invasion	Positive	75 (86%)	27 (90%)	0.59	63 (88%)	39 (87%)	0.90	4 (80%)	98 (88%)	0.62
pT	1 + 2	47 (54%)	16 (53%)	0.95	40 (56%)	23 (51%)	0.64	2 (40%)	61 (54%)	0.53
	3 + 4	40 (46%)	14 (47%)		32 (44%)	22 (49%)		3 (60%)	51 (46%)	
pN	0	53 (61%)	10 (33%)	**0.009**	44 (61%)	19 (42%)	**0.046**	2 (40%)	61 (54%)	0.53
	1	34 (39%)	20 (67%)		28 (39%)	26 (58%)		3 (60%)	51 (46%)	
pM	0	85 (98%)	30 (100%)	0.40	71 (99%)	44 (98%)	0.74	5 (100%)	110 (98%)	0.76
	1	2 (2.0%)	0 (0.0%)		1 (1.4%)	1 (2.2%)		0 (0.0%)	2 (1.8%)	
pStage	I + II	48 (55%)	15 (50%)	0.62	41 (57%)	22 (49%)	0.40	2 (40%)	61 (54%)	0.53
	III + IV	39 (45%)	15 (50%)		31 (43%)	23 (51%)		3 (60%)	51 (46%)	

### Validation of PD-L1 expression on tumor cells by utilizing 2 types of monoclonal antibodies

**Figure 2:**
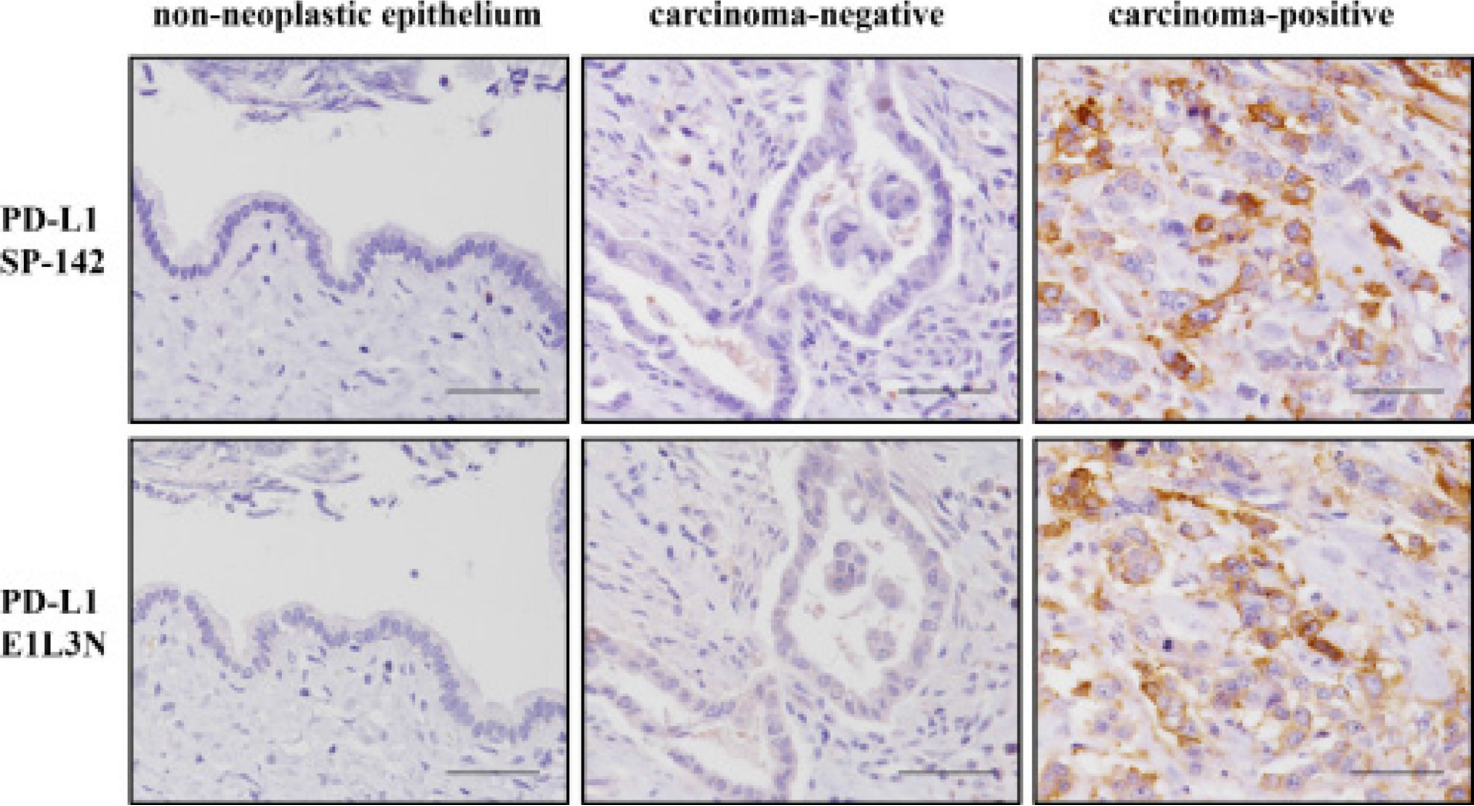
Representative images for immunohistochemical staining of PD-L1 using SP142 (top row) and E1L3N (bottom row) antibodies The carcinoma-negative and -positive images are from the same patient for each antibody. All of the figures are the same magnification (×400). Scale bar, 50 μm.

We immunohistochemically analyzed PD-L1 expression on tumor cells utilizing 2 types of monoclonal antibodies against PD-L1: SP142 and E1L3N (Figure 2). SP142 detected PD-L1 expression in 42 patients (36%), but did not detect any PD-L1 expression in the tumor cells of 75 patients (64%) (Supplementary Figure 1A). When PD-L1 expression as detected by SP142 was analyzed by the H-score, scores ranged between 0 and 140 (median, 0; interquartile range, 0–1.5) (Supplementary Figure 1B). On the other hand, E1L3N detected PD-L1 expression in 53 patients (45%) and did not detect any PD-L1 expression in the tumor cells of 64 patients (55%) (Supplementary Figure 1C). H-scores for PD-L1 expression as detected by E1L3N ranged between 0 and 195 (median, 0; interquartile range, 0–5) (Supplementary Figure 1D).

To explore the best method for evaluating PD-L1 expression compared to methods previously used, we performed multivariate analysis of overall survival according to PD-L1 expression as determined using the 2 types of antibodies (SP142 or E1L3N) with 5 different cutoff values. To determine a cutoff value for the H-score of PD-L1, we used a receiver operating characteristic (ROC) curve. The H-score of PD-L1 as a continuous variable, and median survival as a binary variable, were subjected to ROC analysis. The other four cut-off values used were set based on the percentage of stained tumor cells: 1%, 5%, 10% or 50%. These analyses showed that evaluation using the H-score of SP142 reflected prognosis better than most of the other evaluations (Supplementary Table 3). Therefore, subsequent analyses regarding PD-L1 expression were performed by classifying PD-L1 expression following examination of the H-score of SP142.

### Correlation of the level of PD-L1 expression with TILs and other clinicopathological features

The correlation between PD-L1 expression and clinicopathological factors including TILs is shown in Table 3. Higher expression of PD-L1 was significantly correlated with poorer histological classification (*P* = 0.034). PD-L1 expression was not associated with the infiltration of CD4+, CD8+ or Foxp3+ T lymphocytes.

**Table 3:** PD-L1 expression on tumor cells and its association with clinicopathological features in eCCA and with TILs such as CD4+, CD8+ and Foxp3+ T lymphocytes

		PD-L1 expression		
		High (*n* = 10)	Low (*n* = 107)	*P*
Sex	Male	9 (90%)	84 (79%)	0.39
Age, years	≥71	5 (50%)	54 (50%)	0.98
Tumor size, cm	≥3	3 (30%)	26 (24%)	0.69
Location	Perihilar	4 (40%)	66 (62%)	0.18
	Distal	6 (60%)	41 (38%)	
Histopathological classification	pap+well	0 (0.0%)	34 (32%)	**0.034**
	mod+por	10 (100%)	73 (68%)	
Invasion to hepatic artery	Positive	1 (10%)	4 (3.7%)	0.35
Invasion to portal vein	Positive	2 (20%)	22 (21%)	0.97
Lymphatic vessel invasion	Positive	8 (80%)	74 (69%)	0.47
Venous invasion	Positive	5 (50%)	70 (65%)	0.33
Perineural invasion	Positive	9 (90%)	93 (87%)	0.78
pT	1 + 2	6 (60%)	57 (53%)	0.68
	3 + 4	4 (40%)	50 (47%)	
pN	0	4 (40%)	59 (55%)	0.36
	1	6 (60%)	48 (45%)	
pM	0	10 (100%)	105 (98%)	0.66
	1	0 (0.0%)	2 (1.9%)	
pStage	I + II	4 (40%)	59 (55%)	0.36
	III + IV	6 (60%)	48 (45%)	
Infiltration of CD4+ T lymphocytes	High	7 (70%)	80 (75%)	0.74
Infiltration of CD8+ T lymphocytes	High	6 (60%)	66 (62%)	0.92
Infiltration of Foxp3+ T lymphocytes	High	1 (10%)	4 (3.7%)	0.35

### Correlation of immune responses with EMT-related protein expression

**Figure 3:**
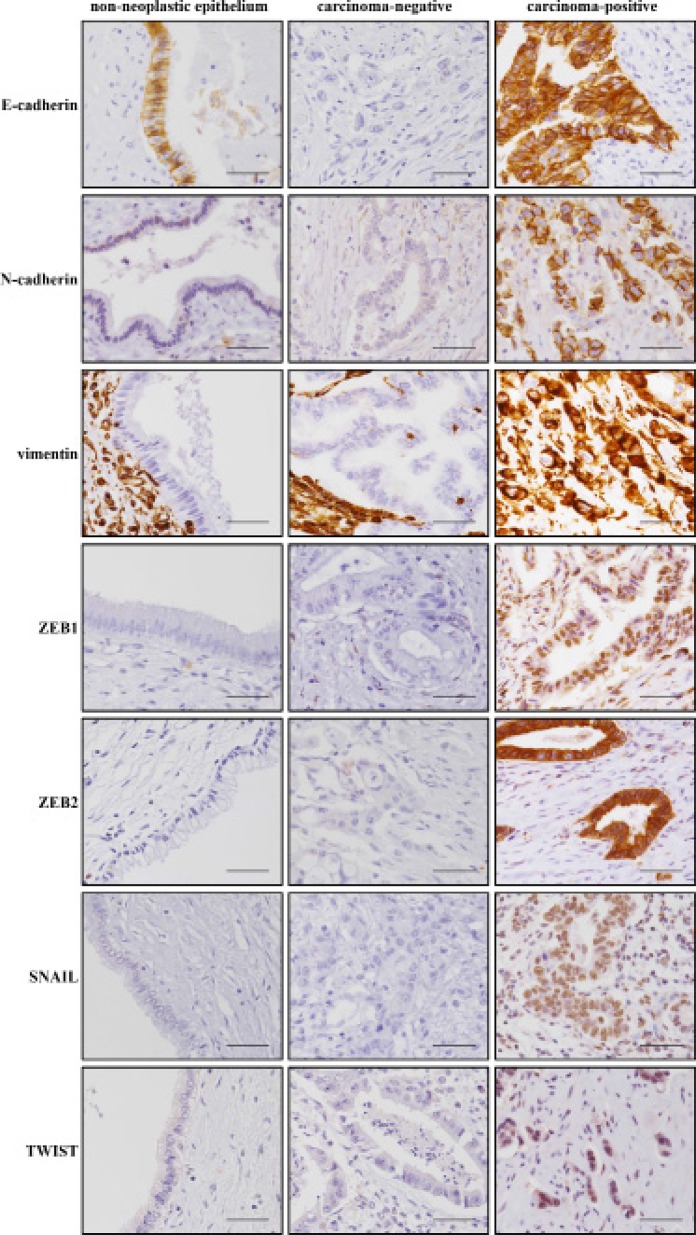
Representative images of immunohistochemical staining for EMT-related proteins E-cadherin, N-cadherin, vimentin, ZEB1, ZEB2, SNAIL and TWIST Each image is from a different patient. All of the figures are the same magnification (×400). Scale bar, 50 μm.

Representative staining patterns of EMT-related proteins are shown in Figure 3. The cutoff values for the EMT related proteins were determined based on the H-scores calculated by ROC analysis to predict patient prognosis. Correlations between TILs and EMT-related proteins were observed (Table 4). High infiltration of CD4+ or CD8+ T lymphocytes was correlated with low SNAIL expression (*P* = 0.016, and *P* = 0.022, respectively). On the other hand, high infiltration of Foxp3+ T lymphocytes was correlated with high vimentin expression (*P* = 0.006). We also examined correlations between PD-L1 expression and EMT-related proteins (Table 4). High expression of PD-L1 was significantly correlated with low expression of E-cadherin (*P* = 0.001), high expression of N-cadherin (*P* = 0.044), high expression of vimentin (*P* < 0.001) and high expression of ZEB1 (*P* = 0.036).

**Table 4:** EMT-related protein expression and its association with TILs such as CD4+, CD8+ and Foxp3+ T lymphocytes and with PD-L1 expression

		Infiltration of CD4+ T lymphocytes			Infiltration of CD8+ T lymphocytes			Infiltration of Foxp3+ T lymphocytes			PD-L1 expression		
		High (*n* = 87)	Low (*n* = 30)	*P*	High (*n* = 45)	Low (*n* = 72)	*P*	High (*n* = 5)	Low (*n* = 112)	*P*	High (*n* = 10)	Low (*n* = 107)	*P*
E-cadherin	High	47 (54%)	12 (40%)	0.19	39 (54%)	20 (44%)	0.31	1 (20%)	58 (52%)	0.16	0 (0.0%)	59 (55%)	**0.001**
N-cadherin	High	14 (16%)	6 (20%)	0.62	10 (14%)	10 (22%)	0.24	2 (40%)	18 (16%)	0.16	4 (40%)	16 (15%)	**0.044**
Vimentin	High	6 (6.9%)	3 (10%)	0.58	6 (8.3%)	3 (6.7%)	0.74	2 (40%)	7 (6.3%)	**0.006**	5 (50%)	4 (3.7%)	**<0.001**
ZEB1	High	26 (30%)	10 (33%)	0.72	22 (31%)	14 (31%)	0.95	2 (40%)	34 (30%)	0.65	6 (60%)	30 (28%)	**0.036**
ZEB2	High	53 (61%)	21 (70%)	0.37	48 (67%)	26 (58%)	0.33	2 (40%)	72 (64%)	0.27	6 (60%)	68 (64%)	0.82
SNAIL	High	33 (38%)	19 (63%)	**0.016**	26 (36%)	26 (58%)	**0.022**	4 (80%)	48 (43%)	0.10	6 (60%)	46 (43%)	0.30
TWIST	High	3 (3.5%)	1 (3.3%)	0.98	2 (2.8%)	2 (4.4%)	0.63	0 (0.0%)	4 (3.6%)	0.67	0 (0.0%)	4 (3.7%)	0.53

### Overall survival associated with immune response and EMT in patients with eCCA

Overall survival according to clinicopathological features including TILs, PD-L1 expression and EMT-related protein expression was examined (Table 5). Univariate analysis of overall survival identified histopathological classification (*P* = 0.032), venous invasion (*P* = 0.024), T (*P* = 0.031), N (*P* = 0.001) and M (*P* = 0.001) classification, the infiltration of CD4+ lymphocytes (*P* = 0.009), and PD-L1 (*P* < 0.001), E-cadherin (*P* = 0.033), N-cadherin (*P* = 0.002) and vimentin (*P* = 0.024) expression as significant prognostic indicators. Multivariate analysis using Cox regression modeling showed that the infiltration of CD4+ T lymphocytes (HR = 0.61; 95% CI = 0.38–1.00; *P* = 0.049), the expression of PD-L1 (HR = 4.27; 95% CI = 1.82–9.39; *P* = 0.001) and the expression of N-cadherin (HR = 2.20; 95% CI = 1.18–3.92; *P* = 0.015) were independent prognostic factors.

**Table 5:** Analysis of prognostic factors for survival in eCCA using Cox proportional hazard modeling

			Survival (%)		Univariate	Multivariate	
		*n*	3-year	5-year	*P*	Hazard ratio (95%CI)	*P*
Sex	Male	93	42	31	0.49		
	Female	24	33	17			
Age, years	<71	58	39	25	0.57		
	≥71	59	42	32			
Tumor size, cm	<3	88	42	32	0.10		
	≥3	29	36	18			
Location	Perihilar	70	49	31	0.13		
	Distal	47	28	24			
Histopathological classification	pap + well	34	62	41	**0.32**	1.07 (0.66–1.78)	0.78
	mod + por	83	32	24			
Invasion to hepatic artery	Negative	112	42	30	0.89		
	Positive	5	20	20			
Invasion to portal vein	Negative	93	44	32	0.14		
	Positive	24	29	13			
Lymphatic vessel invasion	Negative	35	51	31	0.57		
	Positive	82	36	27			
Venous invasion	Negative	42	57	43	**0.024**	1.49 (0.94–2.39)	0.087
	Positive	75	31	20			
Perineural invasion	Negative	15	47	27	0.49		
	Positive	102	40	29			
pT	1 + 2	63	49	36	**0.031**	1.50 (0.96–2.33)	0.076
	3 + 4	54	30	19			
pN	0	63	54	38	**0.001**	1.49 (0.93–2.38)	0.099
	1	54	25	17			
pM	0	115	42	30	**0.001**	4.62 (0.66–20.0)	0.11
	1	2	0	0			
Infiltration of CD4+ T lymphocytes	Low	30	14	14	**0.009**	0.61 (0.38–1.00)	**0.049**
	High	87	49	33			
Infiltration of CD8+ T lymphocytes	Low	45	30	21	0.18		
	High	72	47	33			
Infiltration of Foxp3+ lymphocytes	Low	112	42	30	0.10		
	High	5	0	0			
PD-L1 expression	Low	107	45	32	**<0.001**	4.27 (1.82–9.39)	**0.001**
	High	10	0	0			
E-cadherin expression	Low	58	32	19	**0.033**	0.71 (0.45–1.12)	0.14
	High	59	49	37			
N-cadherin expression	Low	97	47	32	**0.002**	2.20 (1.18–3.92)	**0.015**
	High	20	10	10			
Vimentin expression	Low	108	44	31	**0.024**	0.80 (0.30–1.93)	0.63
	High	9	11	11			

## DISCUSSION

Over the past few years, the tumor microenvironment has been intensively investigated, with a focus on the tumor and the host immune response, particularly from the perspective of immune checkpoint molecules, including PD-L1, PD-1 and CTLA-4 [8, 33, 34]. In the fields of melanoma, lung cancer and bladder carcinoma, which are malignancies representative of cancers involving rich somatic mutation [35], immune checkpoint reagents have been shown to be effective based on evidence derived from randomized controlled studies [36, 37]. In this regard, Le et al. reported that tumors with somatic mutations due to mismatch-repair defects including cholangiocarcinoma, may be susceptible to immune checkpoint blockade [38]. In those studies, the expression level of immune checkpoint molecules was suggested to act as a surrogate marker predicting efficient response to the corresponding reagent and better prognosis. Here, we performed comprehensive immunohistochemical analysis of the local tumor infiltration level of CD4 +, CD8 +, and Foxp3 + T lymphocytes and of PD-L1 expression on tumor cells in resected specimens of eCCA, and we further analyzed their association with EMT-related protein expression.

First, to examine the prognostic significance of PD-L1 expression in eCCA, we validated two different anti-PD-L1 antibodies. The use of the optimal antibody in IHC analyses, demonstrated that high PD-L1 expression in eCCA was an independent poor prognostic factor. The interaction of PD-L1 with its receptor PD-1 is an immune evasion mechanism that suppresses activation of the immune system [16]. Thus, PD-L1 expression has been generally considered to be a poor prognostic factor in several carcinomas [18–20]. However, the prognostic significance of PD-L1 expression in eCCA has been controversial [31, 32]. In this study, we performed multivariate analysis of overall survival in eCCA by using a large database and a better evaluation method of PD-L1 than was used in previous studies. We found that, although the two anti-PD-L1 antibodies displayed similar patterns of staining, the SP142 antibody appeared to stain membranous PD-L1 more specifically than the E1L3N antibody. Mahoney et al. pointed out that, when there is high cytoplasmic PD-L1 staining, discrimination of PD-L1 membranous staining of tumor cells may be less accurate [39]. This difference in the specificity of PD-L1 membranous staining may be the reason why SP142 staining reflected patient prognosis better than E1L3N staining.

Regarding the clinical significance of tumor PD-L1 expression and CD4+ T lymphocyte infiltration in eCCA, our results demonstrated, for the first time to the best of our knowledge, that high PD-L1 expression on the tumor and low tumor infiltration of CD4+ T lymphocytes were independent poor prognostic factors by multivariate analysis, although a correlation between these two factors was not identified. A previous study in eCCA also showed that tumor PD-L1 expression was not correlated significantly with the infiltration of CD8+ T lymphocytes [32]. In terms of the underlying mechanism for the crucial roles of CD4+ T cells, it has recently been reported that CD4+ T cells, especially helper T cells, promote antitumor immunity impacting antigen presentation, co-stimulation, T cell homing, T cell activation, effector function, and memory formation. CD4+ T cells also induce more durable immune mediated tumor control than CD8+ T cells [40, 41]. To further support these critical roles of CD4+ T cells in eCCA, Tran et al. demonstrated the regression of metastatic cholangiocarcinoma following the infusion of CD4+ T cells specific to a neo-antigen (mutated ERBB2IP) [42].

On the other hand, in intrahepatic cholangiocarcinoma (iCCA), a retrospective study showed that the level of CD8+ TILs inversely correlated with PD-L1 expression, and it was hypothesized that this result was due to PD-L1 expression acting as a negative regulator of T lymphocytes [43]. In terms of TILs, a retrospective study has suggested that there is a difference in the immune system between eCCA and iCCA, although these biliary tract cancers are traditionally merged in a clinical context [8]. Therefore, a further study of the correlation between PD-L1 expression and TILs should be conducted. Teng et al. classified cancers into 4 groups on the basis of their PD-L1 status and the presence or absence of TILs [33]. They suggested that the balance between PD-L1 expression and T lymphocyte infiltration is an important factor in determination of the response to immune checkpoint inhibitors. Also, in terms of PD-L1 expression and the expression level of HLA class I, Sabbatino et al. reported significantly better prognosis in the group with high HLA class I expression and low PD-L1 expression in iCCA [18]. Furthermore, Goeppert et al. reported that patients with high HLA class I expression had a higher overall survival probability and that HLA class I expression correlated with the number of TILs in biliary tract cancers [44]. These reports indicated that PD-L1 expression on tumor cells alone does not reflect the tumor microenvironment or the response to immune checkpoint inhibitors.

Regarding the underlying mechanism for the regulation of PD-L1 expression, at least two possibilities have been described. The first mechanism is the upregulation of PD-L1 expression by constitutive oncogenic signaling, such as that seen in Hodgkin lymphoma [22]. The second mechanism is the induction of PD-L1 expression by inflammatory signals such as interferon gamma [21]. In addition to these mechanisms, EMT may correlate with PD-L1 expression [24]. In this study, high PD-L1 expression correlated with low E-cadherin expression, high N-cadherin expression and high vimentin expression, reflecting a mesenchymal phenotype, and also correlated with high levels of expression of the EMT-inducing ZEB1 transcription factor. EMT contributes not only to migratory and invasive properties of the tumor, but also to immunosuppression [23]. A lung cancer study showed that PD-L1 is a downstream target of the interaction between ZEB1 and miR-200, which suppress each other [24]. Among various signaling pathways related to EMT, the PI3K/Akt pathway that regulates miR-200 has been reported to be an important pathway in EMT-induced PD-L1 upregulation in breast cancer [25, 45]. A whole-exome and transcriptome sequencing study of biliary tract cancer showed that a subgroup with an enrichment of hypermutations such as PIK3CA, which encodes PI3K, correlated with expression of immune checkpoint molecules, including PD-L1 [46]. Consistent with those studies, correlations between PD-L1 expression and mesenchymal phenotype have also been reported in several cancers [25, 26, 47–49]. These studies suggested that a subgroup of tumors with an EMT phenotype might be a potential target for treatment using immune checkpoint blockade. Based on these data, it can be presumed that PD-L1 is also regulated by EMT in eCCA. To the best of our knowledge, this study is the first to demonstrate a correlation between PD-L1 expression and EMT-related protein marker expression in eCCA. In terms of why PD-L1 negatively impacts prognosis, past reports have suggested an association between PD-L1 up-expression and down-regulation of T-cell infiltration. Our present data suggest that PD-L1 up-expression could also affect the status of EMT, which enhances the invasion and metastasis of tumor cells resulting in poor prognosis, although a correlation between these two factors was not identified in the present investigation.

We also found that high tumor infiltration of CD4+ and CD8+ T lymphocytes significantly correlated with low tumor expression of SNAIL. This result is consistent with a previous report that SNAIL accelerates cancer metastasis not only through enhanced invasion, but also through induction of immunosuppression such as reduction of TILs by multiple mechanism including immunosuppressive cytokines, regulatory T cells, impaired dendritic cells and cytotoxic T lymphocyte resistance [28].

A key limitation of this study was that the tissue microarray (TMA) method used may not represent the entire tumor specimen due to tissue heterogeneity. Our data showed that a higher number of CD4+ TILs in the invasive front was significantly associated with higher survival rate. These data were consistent with the previous literature on lung cancers and breast cancers, which has reported that high infiltration of CD4+ T cells into the stroma showed better prognosis than that into cancer cell nests [50, 51]. Wakabayashi et al. suggested that CD4+ T cells existing in cancer stroma, but not within cancer nests, might control, or at least reflect, immune responses against cancer cells [50]. A further study using whole-section analysis should be conducted. Additionally, in the present study, we focused on the correlation between PD-L1 expression on tumor cells and TILs, but did not consider PD-1 or CTLA-4 expression on TILs. Therefore, the expression of various immune checkpoint molecules in eCCA should be further studied.

In conclusion, we found that CD4+ T lymphocyte tumor infiltration and PD-L1 expression on tumor cells were independent prognostic factors in eCCA. Furthermore, we provided the first evidence that high tumor PD-L1 expression correlates with a mesenchymal phenotype. Collectively, the present data together with previous findings suggest that the tumor microenvironment in eCCA consists of a complex balance between PD-L1 expression status, levels of TILs and EMT-related protein expression. These findings may help to identify potential biomarkers predictive of not only prognosis, but also therapeutic response in eCCA.

## MATERIALS AND METHODS

### Patient samples

An immunohistochemical evaluation using a TMA method at a single large center was performed. Patients (*n* = 122) underwent surgical resection in the Department of Gastroenterological Surgery II at Hokkaido University Hospital between January 1995 and November 2006 and eCCA tumors were confirmed histopathologically. Five patients were excluded from analysis because insufficient tumor tissue was available for analysis. Ultimately, a total of 117 specimens were evaluated. We categorized eCCA into two groups, perihilar or distal, based on the predominance of the main tumor [2]. All tumors were staged according to the 7th TNM classification system of the Union for International Cancer Control [52]. Study approval was obtained from the Hokkaido University Institutional Review Board (approval number: 015–0501).

### Tissue microarray

TMA blocks were constructed using a manual tissue microarrayer (JF-4; Sakura Finetek Japan, Tokyo, Japan) with a 2.0-mm diameter needle from two representative tumor areas (both the invasive front and the bulk of the tumor) and from one representative area of non-neoplastic bile duct as an internal control. The finalized array blocks were sliced into 4-μm-thick sections and mounted on glass slides.

### Immunohistochemical evaluation

Tissue sections were deparaffinized in xylene and rehydrated through a series of graded ethanol. Heat-induced antigen retrieval was carried out in high-pH antigen retrieval buffer (Dako Cytomation, Glostrup, Denmark). Endogenous peroxidase was quenched with 3% H_2_O_2_ for 5 min. The primary antibodies used are listed in Supplementary Table 4. These sections were visualized using the HRP-labeled polymer method (EnVision FLEX system, Dako Cytomation). Immunostained sections were counterstained with hematoxylin, dehydrated in ethanol, and cleared in xylene.

The analytical validation of the immunohistochemical assay for PD-L1 used in this study was fully evaluated according to the established clinical standard of non-small cell lung cancer. The antigenicity of the formalin-fixed, paraffin-embedded (FFPE) tissue specimen used in this study was assured by vimentin staining. The PD-L1 positive macrophages on each slide were utilized as a positive internal control (Supplementary Figure 2) [53].

The numbers of CD4+, CD8+ and Foxp3+ T lymphocytes were calculated by counting the number of stained infiltrating cells in four high-powered fields in each of the invasive front and the tumor bulk [18]. Semi-quantitative evaluation of PD-L1 or EMT-related protein expression was calculated using H-scores [30, 54]. In brief, the immunohistochemical score was calculated as the product of the percentage of tumor cell positivity and staining intensity (0, none; 1, weak; 2, moderate; 3, intense). The proportion and intensities of tumor cell staining based on the mean of two representative TMA cores were analyzed. The percentage of tumor cells showing different staining intensities was evaluated by two researchers (T.U. and T.M.) who were blinded to the patients’ clinical information. Furthermore, PD-L1 expression was analyzed not only by the H-score, but also by the percentage of stained tumor cells: 1%, 5%, 10% or 50% [20, 55, 56].

### Statistical analysis

A ROC curve was used to determine the cutoff values of continuous variables such as the numbers of CD4+, CD8+ or Foxp3+ T lymphocytes or the H-scores of EMT-related proteins or PD-L1 (Supplementary Figure 1). The values of the percentages of tumor cells with recognized staining alteration as a continuous variable, and survival (alive or dead at the median follow-up time) as a binary variable were subjected to ROC analysis, as previously described [30].

The correlation of each factor such as the tumor infiltration of CD4+, CD8+ or Fop3+ lymphocytes, PD-L1 expression, EMT-related protein expression and clinicopathological factors was analyzed using the Pearson Chi-squared method. Survival was estimated with the Kaplan-Meier method, and survival estimates were compared using the log-rank test. Overall survival was calculated from the date of surgery to the date of death from any cause or last contact/follow-up. Multivariate analysis was conducted using Cox proportional hazards regression modeling. Baseline variables with *P* < 0.05 in univariate analysis were included in multivariate models. The threshold for significance was *P* < 0.05. All statistical analyses were conducted using the JMP for Windows version 12.0 software package (SAS Institute, Cary, NC).

## SUPPLEMENTARY MATERIALS TABLES AND FIGURES



## Abbreviations

PD-1: programmed death 1
PD-L1: programmed cell death ligand 1
eCCA: extrahepatic cholangiocarcinoma
iCCA: intrahepatic cholangiocarcinoma
EMT: epithelial-mesenchymal transition
TILs: tumor-infiltrating T cells
TMA: tissue microarray
