# Supplementary material for: Prognostic impact of programmed cell death ligand 1 (PD-L1) expression and its association with epithelial-mesenchymal transition in extrahepatic cholangiocarcinoma
Source: Oncotarget. 2018 Apr 13;9(28):20034–47. doi: 10.18632/oncotarget.25050 (PMC5929444; doi:10.18632/oncotarget.25050)
Supplement: Supplementary file 1 [file oncotarget-09-20034-s001.pdf]

Prognostic impact of programmed cell death ligand 1 (PD-L1) expression and its association with epithelial-mesenchymal transition in extrahepatic cholangiocarcinoma

SUPPLEMENTARY MATERIALS

Supplementary Table 1:The numbers of CD4+, CD8+ and Foxp3+ TILs were calculated by counting the numbers of stained infiltrating cells in four high-powered fields from each of the invasive front and tumor bulk

	Invasive front			Tumor bulk			*P*
	Median	Range	Interquartile range	Median	Range	Interquartile range	
CD4+ TILs	77	2–384	32–136	59	2–246	33–100	0.15
CD8+ TILs	52	1–212	26–97	55	2–210	28–93	0.94
Foxp3+ TILs	9	0–465	2–21	9	0–145	4–18	0.62

Supplementary Table 2: Multivariate analysis of patient survival based on TILs in the invasive front or tumor bulk.

		Univariate	Multivariate	
		P	Hazard ratio (95%CI)	*P*
CD4+ TILs	Invasive front	**0.009**	0.61 (0.38–1.00)	**0.049**
	Tumor bulk	**0.049**	0.92 (0.59–1.46)	0.73
CD8+ TILs	Invasive front	0.18		
	Tumor bulk	0.059		
Foxp3+ TILs	Invasive front	0.10		
	Tumor bulk	0.055		

Baseline variables showing values of $P < 0.05$ from univariate analysis such as histopathological classification, venous invasion, TNM classification were included in multivariate models.

Supplementary Table 3: Multivariate analysis of patient survival based on several cutoff values of PD-L1 expression.

	Univariate	Multivariate	
	P	**Relative risk (95%CI)**	***P***
SP142			
Proportion cutoff = 1%	0.47		
Proportion cutoff = 5%	0.45		
Proportion cutoff = 10%	**<0.001**	3.78 (1.57–8.35)	**0.004**
Proportion cutoff = 50%	**<0.001**	3.55 (1.00–9.75)	0.050
ROC analysis of H-score	**<0.001**	4.62 (1.82–9.39)	**0.001**
E1L3N			
Proportion cutoff = 1%	0.44		
Proportion cutoff = 5%	0.91		
Proportion cutoff = 10%	0.27		
Proportion cutoff = 50%	**0.012**	1.48 (0.54–3.64)	0.43
ROC analysis of H-score	0.29		

Baseline variables with $P < 0.05$ in univariate analyses such as histopathological classification, venous invasion, TNM classification, the number of CD4+ T lymphocytes, and E-cadherin, N-cadherin and vimentin expression were included in multivariate modeling. Two different clones of anti-PD-L1 antibodies, SP142 and EIL3N, were used for immunohistochemical staining with cutoff values of 1%, 5%, 10% or 50% tumor PD-L1 expression level, and ROC analysis of the H-scores for each antibody were used.

Supplementary Table 4: Antibodies used for immunohistochemistry

Antigen	Clone	Source	Dilution	Retrieval	Staining pattern	Positive cutoff
CD4	SP35	Ventana	ready to use	Tris-EDTA	Membrane	33
CD8	SP57	Ventana	ready to use	Tris-EDTA	Membrane	42
Foxp3	PCH101	eBioscience	1:100	Tris-EDTA	Membrane	97
PD-L1	SP142	SPRING BIOSCIENCE	1:100	Tris-EDTA	Membrane	11 (H-score)
PD-L1	E1L3N	Cell Signaling	1:50	Tris-EDTA	Membrane	6 (H-score)
E-cadherin	4A2C7	Invitrogen	ready to use	Tris-EDTA	Membrane	120
N-cadherin	6G11	Dako	1:50	Tris-EDTA	Membrane	9
Vimentin	V9	Ventana	ready to use	Tris-EDTA	Cytoplasm	60
ZEB1	416A7H10	GenWay	1:20	Tris-EDTA	Nuclear	165
ZEB2	PAb(rabbit)	Novus Biological	1:400	Tris-EDTA	Cytoplasm	30
SNAIL	PAb(goat)	Abcam	1:500	Tris-EDTA	Nuclear	75
TWIST	Twist2C1a	Abcam	1:20	Tris-EDTA	Nuclear	55

A receiver operating characteristic curve was used to determine the cutoff values of continuous variables.

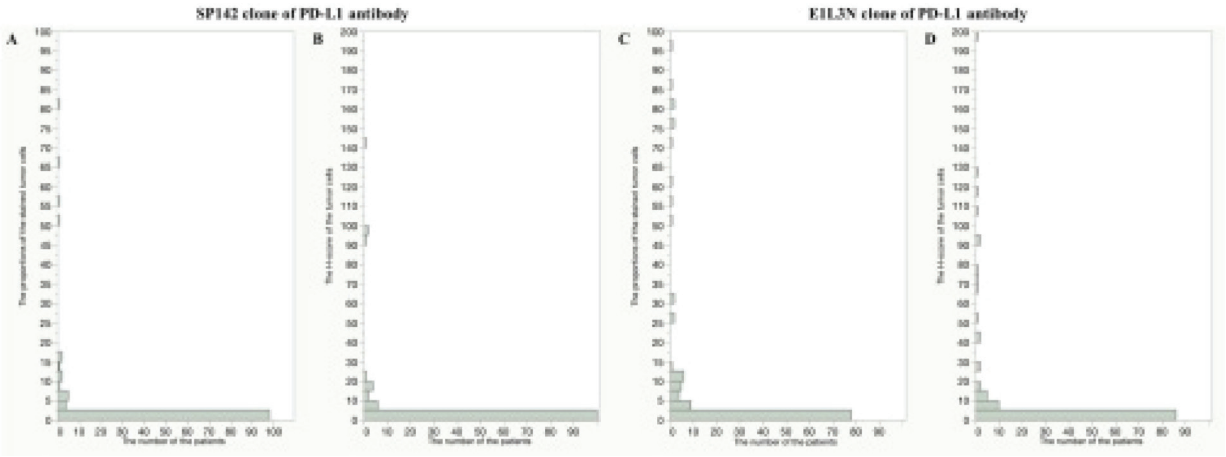

Supplementary Figure 1: The percentage of stained tumor cells and the H-scores of the tumor cells in IHC using the anti-PD-L1 antibodies SP142 and E1L3N. The histograms in (**A**) and (**C**) show the percentage of stained tumor cells when analyzed using SP142 and E1L3N, respectively. The histograms in (**B**) and (**D**) show the H-scores of the tumor cells when analyzed using SP142 and E1L3N, respectively.

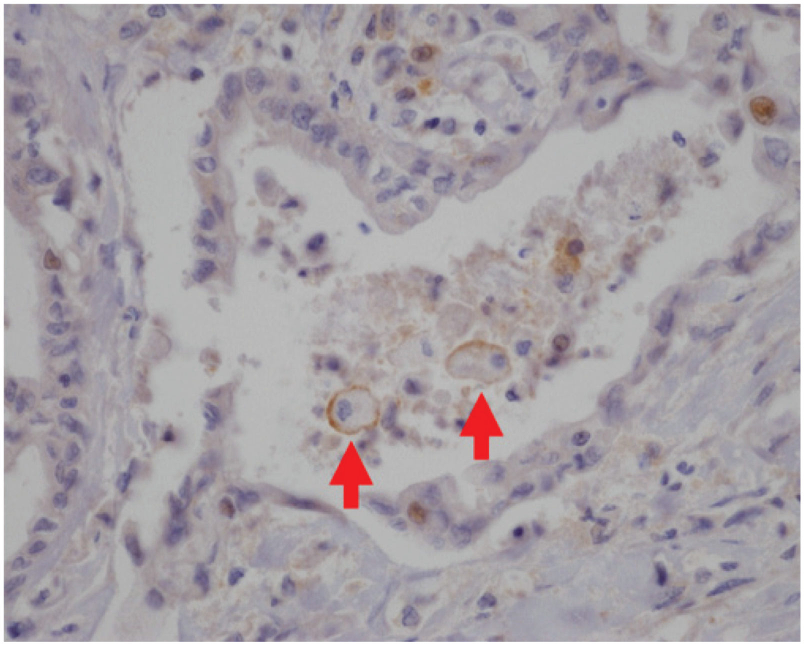

Supplementary Figure 2: Representative immunohistochemical staining of PD-L1 positive macrophages as an on-slide positive internal control (×400).
